# Use of Online Forums for Perinatal Mental Illness, Stigma, and Disclosure: An Exploratory Model

**DOI:** 10.2196/mental.5926

**Published:** 2017-02-20

**Authors:** Donna Moore, Nicholas Drey, Susan Ayers

**Affiliations:** ^1^ Centre for Maternal and Child Health School of Health Sciences City, University of London London United Kingdom; ^2^ School of Health Sciences City, University of London London United Kingdom

**Keywords:** Internet, social stigma, depression, postpartum, mood disorder, female

## Abstract

**Background:**

Perinatal mental illness is a global health concern; however, many women with the illness do not get the treatment they need to recover. Interventions that reduce the stigma around perinatal mental illness have the potential to enable women to disclose their symptoms to health care providers and consequently access treatment. There are many online forums for perinatal mental illness and thousands of women use them. Preliminary research suggests that online forums may promote help-seeking behavior, potentially because they have a role in challenging stigma. This study draws from these findings and theoretical concepts to present a model of forum use, stigma, and disclosure.

**Objective:**

This study tested a model that measured the mediating role of stigma between online forum use and disclosure of affective symptoms to health care providers.

**Methods:**

A Web-based survey of 200 women who were pregnant or had a child younger than 5 years and considered themselves to be experiencing psychological distress was conducted. Women were recruited through social media and questions measured forum usage, perinatal mental illness stigma, disclosure to health care providers, depression and anxiety symptoms, barriers to disclosure, and demographic information.

**Results:**

There was a significant positive indirect effect of length of forum use on disclosure of symptoms through internal stigma, b=0.40, bias-corrected and accelerated (BCa) 95% CI 0.13-0.85. Long-term forum users reported higher levels of internal stigma, and higher internal stigma was associated with disclosure of symptoms to health care providers when controlling for symptoms of depression and anxiety.

**Conclusions:**

Internal stigma mediates the relationship between length of forum use and disclosure to health care providers. Findings suggest that forums have the potential to enable women to recognize and reveal their internal stigma, which may in turn lead to greater disclosure of symptoms to health care providers. Clinicians could refer clients to trustworthy and moderated online forums that facilitate expression of perinatal mental illness stigma and promote disclosure to health care providers.

## Introduction

### Perinatal Mental Illness and Stigma

The term *perinatal mental illnesses* is used throughout this paper to refer to conditions that include depression, anxiety, obsessive compulsive disorder, post-traumatic stress disorder, and puerperal psychosis. Perinatal mental illness is a global health concern [1]. However, prevalence varies depending on a variety of factors such as the instrument used to measure symptoms, developed versus less developed countries, time of evaluation in the gestational or postnatal period, and whether the sample is high risk. Antenatal depression and anxiety occur in approximately 7% to 25% of pregnant women [2-4]. Postnatal depression has a prevalence of 12% to 20% and postnatal anxiety affects 3% to 43% of women [5,6]. Post-traumatic stress disorder occurs in 3.17% of new mothers and can affect up to 15% of women in high-risk groups [7]. Puerperal psychosis occurs in approximately 1-2 per 1000 women [8,9]. Moreover, many women suffer from symptoms associated with mental illness without detection because they do not conform to all the diagnostic criteria [10].

Approximately half the women with perinatal mental illness fail to get professional treatment despite regular contact with health care providers [11]. One reason for this is that the stigma associated with perinatal mental illness can be a barrier to women disclosing and seeking help [12]. External stigma is a negative attitude held by the general public toward an individual or group based on an undesirable quality, for example, mental illness [13]. These negative social stereotypes can lead to labeling the individual as deviant or inferior. Consequently, discriminatory behavior can occur toward the stigmatized group, for example, through social exclusion and marginalization [14]. A UK survey showed that 85% of respondents believed people with mental illness experience stigma and discrimination [15]. There has been noteworthy research and interventions to combat this pervasive social problem; for example, Mind and Rethink Mental Illness developed the “Time to Change” campaign [16].

Internal stigma can occur when stigmatized individuals agree with external stigma and apply it to themselves or they may feel external stigma is unreasonable yet still appraise themselves negatively. There are many detrimental outcomes of internal stigma including lowered self-esteem, lowered life satisfaction, and avoidance of both disclosure and help-seeking behavior [17,18]. High levels of internal stigma have been identified in approximately a third of people with severe mental illnesses [19]. Furthermore, it is important to distinguish between discrimination an individual experiences and external stigma the individual believes others hold without actually experiencing it (known as perceived external stigma). Perceived external stigma correlates with adverse health and mental health outcomes [20,21]. One consequence of perceived external stigma and internal stigma is that they can contribute to stigma associated with disclosure [22]. This paper defines this as “disclosure stigma”: the anticipated negative appraisal and anticipated negative behavior toward the stigmatized individual if he or she chose to disclose to others. This means that stigma could be a key component in an individual’s decision-making process when choosing whether or not to disclose his or her symptoms of mental illness.

### Internet Forums for Stigmatized Mental Illnesses

Internet forums are online discussions where users can have conversations with others by posting messages. They have potential to be an acceptable aid to people with stigmatized conditions as they can use them anonymously, thus circumventing possible negative outcomes associated with stigma [23,24]. Anonymity may also assist online disclosure, which may otherwise be difficult offline [25,26]. Forums for mental health have shown potential for helping people manage or recover from a variety of mental illnesses, in particular, depression [27-29]. They provide an opportunity to connect with similar others and share informational and emotional support [30]. Benefits include social support, communicating experiences with others who share their illness, expressing emotions, group identity, and empowerment and can reduce isolation [29,31-35]. There is some evidence from randomized controlled trials that forum use reduces depressive symptoms [36]. These and other benefits have been reported by both active users (visitors who post on forums) and lurkers (visitors who only read forum messages and do not post), although some evidence suggests lurkers benefit less than active users [37,38]. It is estimated that lurkers make up the majority of forum visitors, with reports of between 45% and 90% of users lurking [39,40].

There is some evidence that engagement with online forums has the potential to reduce stigma [41,42]. Online forums may challenge stigma, help cope with stigma, provide useful tools in testing out disclosing of symptoms and conversing about stigma surrounding mental health. Some research suggests that online forums could challenge stigma through empowerment, giving hope of recovery, and increasing knowledge of health care decisions [29]. Theoretically, all these factors could positively influence disclosure to health care providers and subsequently increase treatment uptake and recovery rates. Disclosure may also be encouraged through social support and posts that direct posters to consult health care providers [30]. Nonetheless, it should be noted that recent research highlights concerns regarding forum use and detrimental outcomes such as social avoidance, Internet addiction, and enabling negative behaviors, such as pro-anorexic forums [43-45].

### Online Forums for Perinatal Mental Illness and Stigma

It is arguable that stigma associated with perinatal mental illness is distinct from mental illness stigma experienced at other times. There are specific concerns related to maternal identity and mental illness such as worries about social services’ involvement, custodial rights, and being judged by others and judging themselves as a “bad mother” [46]. This is complex as some symptoms are highly stigmatized and propagate guilt, shame, and consequent concealment of their illness, for example, suicidal ideation and thoughts of child abuse. Goffman’s (1963) theory of spoiled identity could be applied to understand how women may experience stigma affecting their maternal identity [13]. Many symptoms of perinatal mental illness are seen as incompatible with being a good mother and can exacerbate women’s illness. For example, a woman with postnatal obsessive compulsive disorder might have persistent intrusive thoughts about harming her baby even though she does not act on them. This is a symptom of the illness, but it generates substantial distress and can damage her identity as a mother as these thoughts are not what she thinks a good mother should have.

Indeed, perinatal mental illness stigma could be conceptualized as multifaceted: comprising stigma associated with mental illness and stigma associated with being a mother with a mental illness. This stigma could comprise perceived external stigma, internal stigma, and disclosure stigma. All facets of this stigma could contribute to a woman’s reluctance to disclose symptoms to health care providers [47,48]. It is vital that research and interventions target perinatal mental illness stigma to enable women to disclose, which is the first step to recovery.

One possible avenue for reducing stigma are online forums for perinatal mental illness. There are thousands of online forums for perinatal mental illness with a large flow of traffic indicating that they are highly used [49]. Some evidence highlights that perinatal mental illness forums might provide women with valuable peer support outside the hours of health care provider appointments [50]. Moreover, some research suggests that these forums provide social support that may challenge stigma. One study found an online forum for postnatal depression to be nonjudgmental and posts encouraged users to disclose and seek professional help [51]. Another study documented how online support forums for lesbians with postnatal depression provided social support and a space to communicate stigma [52]. Many women were reluctant to disclose and seek help because of stigma; they were worried about being seen as an unfit mother and concerned their child would be taken away. The dichotomy of “good mother, bad mother” deterred help-seeking behavior; this may be because it contributed to stigma. However, little is known about the relationships between perinatal mental illness forum use, stigma associated with perinatal mental illness, and help-seeking behavior of forum visitors.

A qualitative interview study (N=15) with women who had used forums to assist their recovery from perinatal mental illness explored how women visited forums to reduce their feelings of inadequacy as a mother and perceived stigma from others [53]. Benefits were reported by both active users and lurkers and women valued the anonymity provided by these forums as they could access social support without fear of judgment. Interestingly, most women suggested that forum culture normalized and validated their stigmatized symptoms and unraveled their identity as a “bad mother” with a mental illness from their identity as a “good mother.” Many women felt they were helped by visiting these forums by developing a collective understanding and discourse about their illness. They suggested that this discourse challenged their internal stigma and empowered them to disclose to others offline.

A thematic analysis of posts on an antenatal and postnatal depression forum suggested that use may increase women's disclosure to health care providers, possibly by reducing stigma [22]. Posts provided positive experiences of disclosure to health care providers that challenged women’s concerns about external stigma. Discourse addressed internal stigma by promoting that a “good mother” can have perinatal mental illness and if a woman discloses and gets treatment she is a “good mother.” Forums for postnatal mental illness may therefore provide a place where women can express internal stigma and concerns about external stigma and disclosure stigma. They offer a unique opportunity to anonymously explore sensitive and highly stigmatized issues around their illness with others who may be experiencing the same problems. Potentially, these types of stigma could be challenged by women sharing positive experiences of disclosure. Forum rhetoric could reconstruct the idea of a good mother as compatible with perinatal mental illness, especially if women seek help. Thus, forum use has the potential to increase women's disclosure to health care providers by influencing stigma.

### Aims

**Figure 1:**
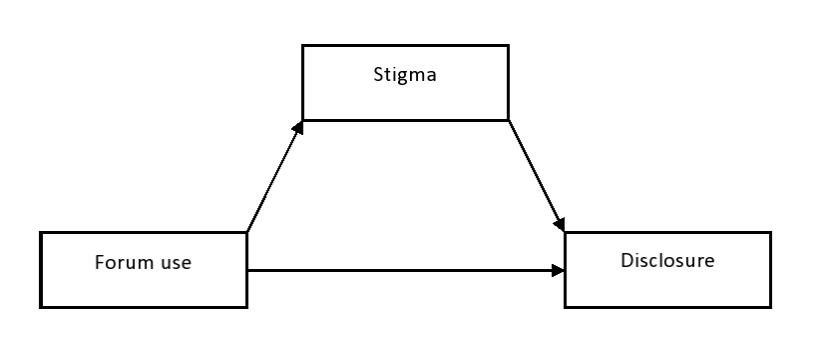
Hypothesis model.

This study aimed to test a model that examined the role perinatal mental illness stigma has in mediating between forum use and disclosure to health care providers. It was hypothesized that perinatal mental illness stigma would mediate between online forum usage and disclosure to health care providers (Figure 1).

## Methods

### Design

A cross-sectional Web-based survey of forum use, stigma, and disclosure in women during pregnancy and up to 5 years after birth who identified themselves as having some level of distress or psychological problems was conducted. Because of the sensitive nature of stigma, the information about the study did not use the term perinatal mental illness as some women might not have wanted to associate with the label; instead, the terms “psychological problems, stress or isolation” were used. Demographic information was collected and symptoms were also measured.

### Participants

Respondents were recruited by advertising on perinatal mental illness websites, motherhood-related websites, Facebook groups, and Twitter. A total of 422 women started the questionnaires via a Web-based survey and 200 had complete answers for the measures used in the model [54]. The survey questions can be found in Multimedia Appendix 1. Inclusion criteria stipulated that women were older than 18 years, were pregnant or had a child younger than 5 years, were a UK resident, considered themselves to be experiencing psychological distress, and had used perinatal mental illness forums.

### Measures

#### City Mental Illness Stigma Scale

Stigma was measured using the City Mental Illness Stigma Scale (City MISS), a 15-item scale that measures the unique stigma women with perinatal mental illness experience (see Multimedia Appendix 1). It was developed from a literature review of perinatal mental illness stigma and tested via a Web-based survey with women with perinatal mental illness (n=279). Factor analysis was used to create the final 15-item scale. The scale accounted for 54.0% of the variance in the sample and had good reliability with Cronbach alphas between .81 and .86 for subscales and an overall alpha of .84 for the total scale indicating high reliability. The City MISS was highly correlated with a reliable measure for mental illness stigma (brief version of the Internalized Stigma of Mental Illness; ISMI-10 scale), which suggested good concurrent validity (*r*_277_= .56, *P*<.001).

Respondents were asked to score the extent to which they agreed with a series of statements measured on Likert scales (1-4): “strongly disagree,” “disagree,” “agree,” and “strongly agree,” with higher scores signifying greater stigma. The scale has a 3-factor structure: the first concerns perceived external stigma, the second internal stigma, and the third disclosure stigma.

The perceived external stigma subscale comprised 6 statements that measured the respondent’s beliefs about what other people think about mothers with psychological problems, for example, “people think mothers with psychological problems will harm themselves.” The internal stigma subscale contained 5 questions that extend the concept of a “spoiled identity”; in this case it was specifically related to the respondent’s identity as a mother, for example, “I have thoughts about leaving my baby” [13].

The disclosure stigma subscale contained 4 questions that measured respondents' anticipated discrimination if they disclosed their symptoms or diagnosis to health care providers. It contained 2 items that measured how they believed others would perceive them if they disclosed their psychological problems, for example, “I worry that if I told a health care provider about my thoughts they would think I am an abusive mother.” It contained 2 items that rated the potential negative consequences of disclosure, for example, “I worry that if I told a health care provider about my psychological problems the social services would get involved.”

#### Hospital Anxiety and Depression Scale

Current affective symptoms were assessed using the Hospital Anxiety and Depression Scale (HADS) [55]. The HADS questionnaire has 14 items, 7 questions to measure depression and 7 questions to measure anxiety; it is widely used to score symptoms for many mental health conditions and is available in many languages [56]. It has good validity and reliability. Many studies from various countries including samples with different health conditions have reported factor analysis results supporting the 2-factor structure: depression and anxiety and significant correlations between the 2 factors [57]. The scale has also shown correlations with other scales that measure depression and anxiety [58].

#### Assessment of Forum Usage, Disclosure, and Demographic Information

Forum usage was measured with questions specifically developed for the study that measured frequency and duration of forum use, frequency of writing posts, and frequency of reading posts. Participation level was classified in accordance with previous literature that defined active and lurker status [59,60]. Women were categorized as active if they started threads or replied to posts and lurker if they did not contribute to forum conversations. Frequency of forum visits and duration of forum use categories were developed by all authors and subject to data analysis several times using different classifications and cutoffs to see if the results were altered. All authors agreed on the final categories based on face validity and categories with approximately equal numbers. Frequency of forum visits was measured as occasional when women reported visiting 3 times or less a month and frequent when reported as once or more a week. Duration of forum use was measured as long-term for members who had been visiting a year or more and short-term if under a year.

Disclosure was ascertained by participants indicating the people they had disclosed symptoms or diagnosis to, for example, a health visitor, family member, midwife. Results were coded as disclosed to a health care provider or not disclosed to a health care provider.

Demographic information was collected about marital status, ethnicity, occupation, education, number of children, and diagnosis.

### Procedure

The survey was pilot-tested with 6 women with children younger than 2 years who had perinatal mental illness; the participants completed the questionnaire online using a mobile phone and all found it acceptable and easy to understand and answer. Ethical approval was given by City, University of London, United Kingdom. Website and forum administrators were contacted to ask for consent to post information about the study and a hyperlink to the survey. The participant information sheet and the first and last pages of the survey urged women to contact their health care provider should they feel upset and provided details of external organizations that offered support. The first page of the survey was the participant information sheet and participants had to click a box to confirm that they consented to the study before they could progress to the survey.

### Data Analysis

Results were included if the respondent had completed all the forum questions and the City MISS questions (n=200). Internet protocol addresses were checked for duplication, but none were identified. The models were analyzed using the statistical software package IBM SPSS 20.0 (IBM Corporation) and the moderation and mediation plug-in PROCESS [61]. Mediation analysis was conducted using the method suggested by Hayes [62]. This method was used because it tests whether there is an indirect effect and has good power because it uses bootstrapping [62,63]. Frequency of forum visits, duration of forum use, and frequency of writing posts were entered as predictor variables. City MISS scores for subscales internal stigma, external stigma, and disclosure stigma were entered as mediators. The outcome variable was disclosure of symptoms to a health care provider. HADS scores were entered as a covariate to account for potential confounding effects on the relationships between variables. The bootstrapping method was used to test the potential mediation effect using 1000 iterations. This method was chosen as it allows for multiple mediators, controls for the effects of covariates, has a higher power than the traditional Sobel test, and reduces the possibility of a type I error. The bootstrapping procedure computes the confidence intervals (CIs) for the indirect path. The null hypothesis is that the indirect path does not significantly differ from zero, so if the CIs do not include zero then the null hypothesis can be rejected and the indirect effect is significant when *P*<.05. Qualitative answers regarding disclosure barriers were transferred to the NVivo 10 (QSR International) software package and coded for external stigma, internal stigma, and disclosure stigma [64].

## Results

### Sample Characteristics

Participant characteristics are provided in Table 1. Most participants classed themselves as white (n=191) and married or living with a partner (n=133, n=48). More than 60.0% (125/200) were educated to degree level or above and most had 1 child (n=139). The majority of women had a diagnosis of one or more postnatal mental illnesses (142/200, 71.0%), and 23.0% (46/200) of women reported postnatal mental illness but had not sought a professional diagnosis. The remaining women had a diagnosis of antenatal mental illness (3/200, 1.5%) or both antenatal and postnatal mental illness (9/200, 4.5%). On the basis of the HADS measure completed during this study (n=200), 66.5% (133/200) of the women had moderate or severe anxiety and 34.5% (69/200) had moderate or severe depression. Forum use characteristics are presented in Table 2. Women were almost evenly categorized as frequent or occasional users and long-term or short-term users (n=108, n=92). There were nearly 3 times as many active users than lurkers (n=156, n=44).

### Correlational Analysis

Relationships between variables are presented in Table 3. The length of forum use was significantly correlated to internal stigma score (subscale of City MISS). Frequency of forum visits was significantly correlated to participation level. Total stigma score was significantly correlated to internal stigma, external stigma, and disclosure stigma (stigma subscales of City MISS). Internal stigma score was significantly correlated to disclosure to a health care provider. Total affective symptoms score was significantly correlated to total stigma score, internal stigma, external stigma, and disclosure stigma. Total affective symptoms score was the only variable that had significant correlations with the model variables, that is, all mediator variables. Therefore, total affective symptoms score was entered as a covariate in the following analyses.

### Stigma Mediating the Relationship Between Forum Use and Disclosure

**Figure 2:**
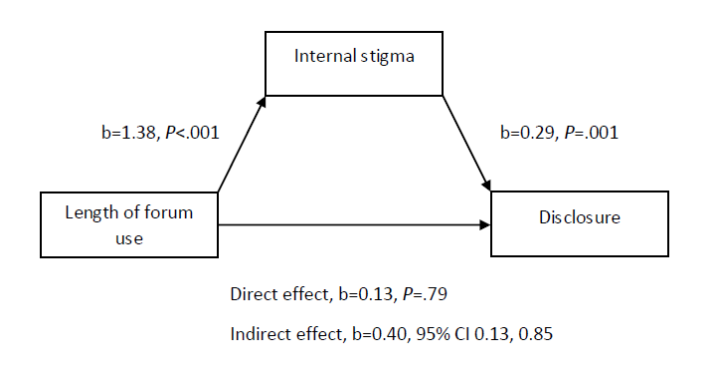
Model with statistics.

There was a significant indirect effect of length of forum use on disclosure of symptoms to a health care provider through internal stigma, b=0.399, bias-corrected and accelerated (BCa) CI 0.133-0.846. There were no other indirect effects between variables and there was no direct effect between forum variables and disclosure, b=0.133, *P*=.79 (Figure 2).

**Table 1:** Sample demographic characteristics.

Characteristics	Subcategories	n (%) (N=200^a^)
Marital status	Single	12 (6.0)
	Married	133 (66.5)
	Living with partner	48 (24.0)
	Separated	3 (1.5)
	Other	2 (1.0)
Ethnicity	White	191 (95.5)
	Mixed or multiple ethnic groups	5 (2.5)
	Other	2 (1.0)
Occupation	Employed	104 (52.0)
	Self-employed	17 (8.5)
	Out of work	9 (4.5)
	Homemaker	47 (23.5)
	Student	11 (5.5)
	Unable to work	10 (5.0)
Education	GCSE^b^	17 (8.5)
	A Levels	30 (15.0)
	Trade or vocational training	26 (13.0)
	Bachelor’s degree	89 (44.5)
	Master’s degree	34 (17.0)
	Doctoral degree	1 (0.5)
Number of children	1	139 (69.5)
	2	45 (22.5)
	3	6 (3.0)
Diagnosis	Antenatal depression	2 (1.0)
	Antenatal depression and anxiety	1 (0.5)
	Antenatal depression and postnatal depression	9 (4.5)
	Postnatal depression	53 (26.5)
	Postnatal anxiety	9 (4.5)
	Post-traumatic stress disorder (PTSD)	23 (11.5)
	Postnatal depression and anxiety	23 (11.5)
	Postnatal depression and PTSD	19 (9.5)
	Three or more postnatal mental illnesses	7 (3.5)
	Puerperal psychosis	3 (1.5)
	Other postnatal mental illness	5 (2.5)
	No diagnosis sought	46 (23.0)

^a^The total number of participants in demographic categories does not add up to 200 as not all participants completed the demographic questions.

^b^GCSE: General Certificate of Secondary Education.

**Table 2:** Sample forum use characteristics.

Variable	Category	n (%) (N=200)
Frequency of visits	Frequent	108 (54.0)
	Occasional	92 (46.0)
Participation level	Active	156 (78.0)
	Lurker	44 (22.0)
Duration of use	Long-term	108 (54.0)
	Short-term	92 (46.0)

**Table 3:** Bivariate correlations of dependent variables, independent variables, mediators, and control variable.

Variable	1	2	3	4	5	6	7	8	9
1. Disclosure	1	.060	−.004	.045	.138	.230^c^	.074	−.019	.019
2. Length of use		1	−.087	.043	.150^d^	.180^d^	.064	.087	−.020
3. Frequency of visits			1	.285^c^	.088	.065	.031	.111	.066
4. Participation				1	.058	.095	.061	−.041	.032
5. Total City MISS^a^					1	.751^c^	.766^c^	.744^c^	.423^c^
6. Internal stigma						1	.299^c^	.331^c^	.393^c^
7. External stigma							1	.434^c^	.222^c^
8. Disclosure stigma								1	.339^c^
9. HADS^b^									1

^a^City MISS: Mental Illness Stigma Scale.

^b^HADS: Hospital Anxiety and Depression Scale.

^c^Correlation is significant at the .01 level (two-tailed).

^d^Correlation is significant at the .05 level (two-tailed).

## Discussion

### Principal Findings

This study tested a model based on previous research that proposed perinatal mental illness stigma mediates between forum use and disclosure. There was a significant indirect effect of length of forum use on disclosure of symptoms to health care providers through internal stigma. Long-term forum users reported higher internal stigma scores and higher internal stigma was associated with disclosure to a health care provider. There was no indirect effect of perceived external or disclosure stigma, nor was there a direct effect between length of forum use and disclosure. Qualitative findings support this as many women reported that external stigma, internal stigma, and disclosure stigma specific to perinatal mental illness were barriers to their disclosing to health care providers.

There was theoretical reason to believe that increased forum use would decrease stigma and subsequently increase disclosure to health care providers. Also, social support offered by forum members might be empowering and play a role in disclosure. Results unexpectedly indicated that forum use was associated with increased reported internal stigma. There are a number of possible explanations for this. It is plausible that visiting forums might have adversely affected internal stigma or it may be that women with higher internal stigma use forums for longer. However, this study advocates an alternative explanation of the findings and proposes that forum messages encouraged these women to recognize their feelings of internal stigma, and this increased their self-awareness, enabling them to make a fuller disclosure when completing the stigma questionnaire. Previous research suggests that forums may provide a place where women can explore their feelings, and this may have meant that implicit feelings of internal stigma could have become explicit [22]. Also, women may have overcome barriers to revealing this internal stigma through forum benefits such as social support, identification with the forum group, and an alternative perspective of perinatal mental illness that shifted their “spoiled identity” to an empowered identity as a good mother despite having a mental illness.

Interestingly, there was no direct effect between forum use variables and disclosure. Current research suggests that mediation can occur without results showing a direct effect [65,66]. It is possible that other factors omitted from the analyses may have weakened the direct effect, also known as suppressor variables. Possible suppressor variables are individual differences such as personality traits, experience of discrimination, and social support. Furthermore, there may have been an overrepresentation of a subset of women who do not have a significant relationship between the predictor and outcome variables, and thus a direct effect would have been neutralized. Future research could measure potential suppressor variables to explore this intricate relationship.

### Limitations

Caution should be exercised when considering generalization from this sample as there are a number of characteristics that might not be true of the general population of forum users. First, the women were mostly white and educated to A Level or above. There was also an overrepresentation of active users, possibly because active users have a more vested interest in forum research or they have different traits that might make them more likely to post on forums and participate in Web-based surveys [45]. The majority of women had experienced symptoms in the postnatal period, and thus findings might not extend to antenatal populations. The model could benefit from future modifications such as including a measure of whether women had experienced discrimination when disclosing, such as “health professional stigma” [67]. Similarly, there was no measure of other social media use that may affect stigma levels and have further implications for disclosure behavior. The type of forum or combination of forums women used may have had an impact on the results, for example, if the forum was moderated or unmoderated. The study cannot determine the direction of causality because it was cross-sectional. It may therefore be that women who disclosed may be more likely to visit forums rather than forum use in itself increasing disclosure behavior. Future research should use experimental and longitudinal designs to be able to ascertain the direction of this potential effect and include a measure of forum type. Future research could test the theories suggested as part of prevention and intervention studies. It might also be beneficial to include other means of social support into the model.

### Comparison With Prior Work

This study supports and extends previous research that suggests forum use may affect internal stigma and in turn lead to disclosure; however, this relationship may be more complex than our initial model proposed [41,42]. Interpretation of the findings suggests that perceived external stigma and disclosure stigma do not mediate between forum use and disclosure. This is surprising because prior studies showed that perinatal mental illness forum messages are pro-disclosure and supply positive experiences with health care providers [22,51,52].

Another consideration is that forums differ in their posts concerning health care providers. Recent research has highlighted that some forums include many negative experiences with illness symptoms and experiences with health care providers [68]. Thus, some forums may contain conversations that reduce disclosure stigma and external stigma, and others, albeit inadvertently, may increase external stigma and disclosure stigma. Women in this sample may have been visiting a mixture of these types of forums and they may have had different effects on perceived external stigma and disclosure stigma. Therefore, future research should not dismiss exploration of external stigma and disclosure stigma in forum use.

Qualitative findings confirm that stigma is a major barrier to disclosure and details the complex relationship between maternal identity and internal stigma, external stigma, and disclosure stigma [22]. Other studies advocated a negative effect on disclosure from an overreliance on forums, but this was not suggested in our findings [44,45]. However, it may be worth investigating whether there are forums that maintain or generate stigma, for example, through providing negative experiences with health care providers, such as a forum for birth trauma.

### Conclusions

Findings suggest internal stigma of perinatal mental illness mediates between forum use and disclosure. Using forums may provide valuable social support and improve women’s disclosure to health care providers through enabling expression of their internal stigma. Theoretical reasons for this have been discussed; in particular, it is suggested that women can explore their feelings, so they become more aware of their internal stigma and are empowered to express these feelings. Furthermore, women may be enabled to disclose by reconciling the negative impact of internal stigma on their maternal identity by agreeing with forum discourse that promoted the idea that one can be a good mother despite having perinatal mental illness. Future work could test these theories and continue to strengthen knowledge of perinatal mental illness, forum use, stigma, and disclosure behavior. Clinicians could refer clients to trustworthy and moderated online forums that facilitate expression of perinatal mental illness stigma and promote disclosure to health care providers.

## Appendix

Multimedia Appendix 1 Web-based survey questions.

## Abbreviations

BCa: bias-corrected and accelerated
City MISS: City Mental Illness Stigma Scale
HADS: Hospital Anxiety and Depression Scale
