# Supplementary material for: Use of Online Forums for Perinatal Mental Illness, Stigma, and Disclosure: An Exploratory Model
Source: JMIR Ment Health. 2017 Feb 20;4(1):e6. doi: 10.2196/mental.5926 (PMC5339438; doi:10.2196/mental.5926)
Supplement: Multimedia Appendix 1 [file mental_v4i1e6_app1.pdf]

Appendix 1. Online survey questions

Online survey questions

Section 1 About you

Please rate the level of psychological problems, stress and/or isolation you had in pregnancy and/or after birth?

None ☐

Mild ☐

Moderate ☐

Severe ☐

Have you talked to any of the following people about these feelings? Please tick all that apply

Partner ☐

Family ☐

Friends ☐

Family Doctor/GP ☐

Midwife ☐

Online forum users ☐

Health visitor ☐

Counsellor/therapist ☐

other ☐
please state

Have you been diagnosed by a healthcare professional as having a psychological problem during pregnancy and/or after birth?

Yes ☐

No ☐
If yes, please state what they said the problem was

Are there any things that make it difficult for you to talk to healthcare providers about how you feel/felt during pregnancy and/or after birth?

No ☐

Yes ☐
if yes please describe

Are you receiving or have you received any of the following treatments in the time around the birth of your child? please tick all that apply

Mother and baby unit ☐

Counselling ☐

Medication ☐

Cognitive Behavioural Therapy (CBT) ☐

Online self-help ☐

other ☐

please state

<u>Section 2 What do you think about psychological problems during pregnancy and after birth?</u>

The City Mental Illness Stigma Scale (Moore, Ayers & Drey)

We use the term “psychological problems” in this questionnaire to refer to all sorts of distress mothers might experience after having a baby, for example mood problems, depression, anxiety, trauma and obsessive compulsive disorder. Please think of psychological problems to mean whatever you feel comfortable with. We appreciate that some questions may be of a sensitive nature and we thank you for your honesty in helping us better understand how you are feeling.

Please select the amount you agree or disagree with each of the following statements.

	Strongly disagree	Disagree	Agree	Strongly agree
1. I can’t cope as well as I’d like with my baby	1	2	3	4
2. I have thoughts of hurting myself	1	2	3	4
3. I have thoughts of killing myself	1	2	3	4
4. I have thoughts about leaving my baby	1	2	3	4
5. My psychological problems have meant I have lost time with my baby	1	2	3	4
6. People think mothers with psychological problems can’t cope with their babies	1	2	3	4
7. People think mothers with psychological problems don’t love their babies	1	2	3	4
8. People think mothers with psychological problems will harm their babies	1	2	3	4
9. People think mothers with psychological problems will harm themselves	1	2	3	4
10. People think mothers with psychological problems will kill themselves	1	2	3	4
11. People think mothers with psychological problems are abnormal	1	2	3	4
12. I do not want people to know I have psychological problems as they may think I’m a bad mother	1	2	3	4
13. I worry that if I told a healthcare provider about my psychological problems the social services would get involved	1	2	3	4
14. I worry that if I told a healthcare provider about my psychological	1	2	3	4

problems my baby would be taken away				
15. I worry that if I told a healthcare provider about my thoughts they would think I am an abusive mother	1	2	3	4

Section 3 What do you think about mental illness in general?

Brief Version of the Internalized Stigma of Mental Illness (ISMI) Scale (Boyd, Otilingam & DeForge, 2014)

Section 4 About using online forums

How often do you visit forums that discuss mental health in pregnancy or after birth? For example, depression after childbirth, upsetting feelings around birth.

daily ☐

weekly ☐

monthly ☐

never ☐

Please list the forum(s) that you use most often about these issues?

When did you start using these forum(s)?

A week ago ☐

A month ago ☐

6 months ago ☐

A year ago ☐

Over a year ago ☐

How often do you read posts on these forums?

daily ☐

weekly ☐

monthly ☐

never ☐

How often do you start a conversation/thread on these forums?

daily ☐

weekly ☐

monthly ☐

never ☐

How often do you reply to conversations/thread posted by other members?

daily ☐
weekly ☐
monthly ☐
never ☐

What are the main reasons you visit these forums?

Section 5 How you feel now

Hospital Anxiety and Depression Scale (Zigmond & Snaith, 1983)

Part 6 Tell us about yourself

What is your age?

What is your child's/children's date of birth?

Which category best describes you?
White ☐
Hispanic or Latino ☐
Black or African American ☐
Native American or American Indian ☐
Asian / Pacific Islander ☐
Other please state ☐

What is your country of residence?

What is your current marital status?

Single, never married ☐
Married ☐
Living with partner ☐
Divorced ☐

Widowed ☐
Separated ☐
other ☐

Are you currently?

Employed for wages ☐

Self-employed ☐

Out of work and looking for work ☐

Out of work but not currently looking for work ☐

A homemaker ☐

A student ☐

Military ☐

Unable to work ☐

What is the highest level of education you have competed?

GCSEs ☐

A levels ☐

Trade/technical/vocational training ☐

Bachelor's degree ☐

Master's degree ☐

Doctorate degree ☐
